# Supplementary material for: Automated detection scheme for acute myocardial infarction using convolutional neural network and long short-term memory
Source: PLoS One. 2022 Feb 25;17(2):e0264002. doi: 10.1371/journal.pone.0264002 (PMC8880846; doi:10.1371/journal.pone.0264002)
Supplement: Attachment — Submitted filename: Response_R1_20210717.docx [file pone.0264002.s001.docx]

PONE-D-21-16074
Title: Automated detection scheme for acute myocardial infarction using convolutional neural network and long short-term memory

**Dear Editor and Reviewers:**

Thank you for reviewing our manuscript. We have revised it based on your comments. Our responses are provided below.

**Response to Reviewer #1:**

**Comment 1:**

Spell out each acronym the first time used in the Abstract as well as the body of the paper.

**Response:**

Thank you for your comments. We have checked all the terms.

**Comment 2:**

Make sure all figures and tables are referred to in the text.

**Response:**

Based on your comment, the manuscript was checked to make sure that the tables in the figures were cited correctly.

**Comment 3:**

There are many typos and grammatical mistakes in the entire paper.

**Response:**

Thank you for your comments. The entire text was checked and proofread again by a native English speaker.

**Comment 4:**

There must be column names in the tables with all borders.

**Response:**

Thank you for your comments. We have checked the table and confirmed that the column names are marked. In addition, there was a slight error in the style of the table, which has been corrected.

**Comment 5:**

All the key terms of the equations must be mentioned.

**Response:**

Thank you for your comments. We have checked the variables and terms that appear in the formulas. There were some terms that were not fully explained, so we have corrected them. (LSTM networks, P.11-12, Lines 189, 204, 208, 211, 214)

**Comment 6:**

Qualities of figures are not good.

**Response:**

Thank you for your comments. We have uploaded the file with the original images. Furthermore, we have uploaded the original figure data to public repository at the following URL:

https://doi.org/10.6084/m9.figshare.14999154.v1.

**Comment 7:**

Relevant literature review of latest similar research studies on the topic at hand must be discussed.

**Response:**

Thank you for your comments. We have researched the latest similar studies on the topic and added them to the introduction section.

*” Kusunose et al. conducted a study on a novel detection method using deep learning techniques for abnormal regional wall motion noted on echocardiography. Multiple deep learning models were utilized to classify echocardiography into routine normal cases and those with abnormal regional wall motion. They adopted the ROC curve as an evaluation method and compared the classification accuracy of the clinical technologists to that of the proposed deep learning model. This comparison resulted in the AUC of the proposed method (0.97) and the AUC of the clinical technologists (0.95) being virtually equivalent, confirming the effectiveness of the proposed method. However, the study had a few limitations. The first limitation was the use of short-axis echocardiogram views only at the level of the papillary muscle. Second, the deep learning input data were limited to only three images per case: end-diastolic, mid-systolic, and end-systolic. Therefore, we attempted to detect AMI by analyzing the wall motion temporal changes and inputting the echocardiograph of one cardiac cycle into deep learning, which can then analyze time series. In addition to the short-axis papillary muscle (PM) level view, we used another view that is easy to use, the left ventricular long axis view, to detect AMI. We believe this method could be integral to the development of detection support technology so that non-specialist physicians in their clinics can accurately detect an AMI.”*　(Introduction, P.4-5, Lines 74-88)

**Comment 8:**

The summary at the end of the literature review should be focused on the limitations of related work.

**Response:**

Thank you for your comments. As mentioned in Comment 7, we focused on the limitation of previous studies and noted my motivation for this study. (Introduction, P.4-5, Lines 74-88)

**Comment 9:**

The authors should clearly mention all parameters used to evaluate the performance of the classifiers.

**Response:**

Thank you for your comments. For the hyperparameters of LSTM, we set the learning rate to 1 × ${10}^{-5}$, the number of epochs to 50, the batch size to 30, and the input data size to 4096 × 30. For the hyperparameters of ANN, we set the learning rate to 1 × ${10}^{-5}$, the number of epochs to 50, the batch size to 30, and the input data size to 4096 × 30. The hyperparameters are also described in the LSTM networks section on p. 13, Comparison with conventional artificial neural networks on p. 15. (LSTM networks, P.13 Lines 227-228 & Comparison with conventional artificial neural network, P.15 Lines 253-255)

**Comment 10:**

- Authors can add below references:

1) Rehman, A., Rehman, S. U., Khan, M., Alazab, M., & Reddy, T. (2021). CANintelliIDS: Detecting In-Vehicle Intrusion Attacks on a Controller Area Network using CNN and Attention-based GRU. IEEE Transactions on Network Science and Engineering.

2) Abbas, S., Jalil, Z., Javed, A. R., Batool, I., Khan, M. Z., Noorwali, A., ... & Akbar, A. (2021). BCD-WERT: a novel approach for breast cancer detection using whale optimization based efficient features and extremely randomized tree algorithm. PeerJ Computer Science, 7, e390.

**Response:**

Thank you for providing useful literature. It has been cited as a reference for this study. (References, P.26-27, lines 434-436 & 439-441)

**Response to Reviewer #2:**

**Comment 1:**

What are the limitations of existing works that motivated the current research?

**Response:**

Thank you for your comments. The first limitation was the use of short-axis echocardiogram views only at the level of the papillary muscle. Therefore, we attempted to develop a method to aid in the detection of AMI in doctors who are not specialists or doctors in clinics by adding left ventricular LX views, which is easy to draw. This review has also been added to the introduction section on p. 5. (Introduction, P.5, Lines 81-87)

**Comment 2:**

What is the novelty of the work?

**Response:**

Thank you for your comments. The novelty of our method is that we attempted to automatically classify AMI in two types of views: short-axis PM level and left ventricular LX views. In previous studies, the deep learning input data were limited to only three images per case: end-diastolic, mid-systolic, and end-systolic. Therefore, we aimed to analyze and classify myocardial motion in detail, and selected 30 frames of echocardiography for one cardiac cycle to input for deep leaning. The other novelty is that we introduced CNN and LSTM, which can analyze time series, to analyze the heart motion and classify the normal myocardium and AMI cases. We have added these novelties to the introduction section on p. 5. (Introduction, P.5, Lines 81-91)

**Comment 3:**

List out the main contributions of the current work?

**Response:**

Thank you for your comments. Cardiologists and experienced sonographers usually estimate the culprit coronary artery in patients with myocardial infarction using left ventricular long-axis, left ventricular short-axis, apical four-chamber, apical two-chamber, and apical long-axis views. Especially acute anteroseptal infarction is evaluated using left ventricular LX and short-axis PM level views as well as apical four-chamber and apical LX views, which allow observation of the apex. However, inexperienced clinicians, non-cardiologists, residents, and those otherwise unfamiliar with echocardiography may find it difficult to obtain apical four-chamber and apical LX view images with adequate quality. In this study, we detected AMI using short-axis PM and left ventricular LX views, which are the minimum for diagnosis and easy to depict. These results indicate that this method correctly identifies acute anteroseptal infarction with superior accuracy clearly distinguishing it from normal myocardium, and that it can assist non-cardiologists and inexperienced clinical technologists diagnose acute anteroseptal infarction during initial treatment, leading to appropriate treatment of patients with AMI and improvement of their prognosis.

the classification accuracy of LSTM suggests that the image information of one cardiac cycle (consisting of 30 frames) is useful for analyzing myocardial motion, thereby distinguishing AMI from normal myocardium. Unlike ordinary ANNs, LSTMs have a mechanism known as “gates,” optimizing them for time-series information analysis. The results showed that the LSTM can detect AMI on echocardiography with better accuracy than ANN by analyzing time series information, and the superiority of the proposed method using LSTM was confirmed. (Discussion, P.21, Lines 346-357 & P.19, Lines 314-318.)

**Comment 4:**

Summarize the related works in the form of a table.

**Response:**

Thank you for your comments. We have researched the previous studies and added the following table. The table has been added to the Discussion section. (Discussion, P.22, Lines 359-360)

| Author | dataset | purpose | method | performance |
| --- | --- | --- | --- | --- |
| Kusunose et al.[7] | Patient with old myocardial infarction :400  Normal :100 | Detection of regional abnormal wall motion on echocardiography | Classification using CNN | AUC:0.97 |
| Zhang et al.[32] | Patient with myocardial infarction :169  Control:69 | Detection of chronic myocardial infarction on nonenhanced cardiac cine MRI | Classification using region extraction model and LSTM | Sensitivity:0.90  Specificity:0.99  AUC:0.94 |
| Baloglu et al.[33] | Myocardial infarction :148  Normal:52 | Detection of myocardial infarction on 12-lead ECG | Classification using CNN | Overall accuracy:0.9978 |
| Huang et al[9] | Regional wall motion abnormality:947 | Detection of regional abnormal wall motion on echocardiography | Segmentation using U-Net | Dice index:0.756 |
| Vece et al[34] | Patient with takotsubo syndrome :110  Patient with acute myocardial infarction:110 | Detection of Takotsubo cardiomyopathy on echocardiography | Classification using machine learning | AUC:0.801  Overall accuracy:0.745 |
| Shimizu et al[35] | Patient with takotsubo syndrome :50  Patient with acute myocardial infarction:50 | Detection of Takotsubo cardiomyopathy and AMI on echocardiography | Classification using CNN | Mean accuracy:0.748 |
| Shahin et al[36] | 8 of cardio views : 432 | Classification of echocardiographic view types | Classification using CNN and LSTM | Accuracy :0.963 |

**Comment 5:**

Some of the recent works on LSTM and CNN applied in different domains such as the following can be discussed in the paper: "Hand gesture classification using a novel CNN-crow search algorithm, A multidirectional LSTM model for predicting the stability of a smart grid, Image-Based malware classification using ensemble of CNN architectures (IMCEC)"

**Response:**

Thank you for providing useful literature. It has been cited as a reference for this study.

(References, P.25-26, lines 408-410, 411-412, 423-425)

**Comment 6:**

Compare the results obtained with recent state-of-the-art.

**Response:**

Thank you for your comments. An AI-based study on laryngitis pathology classification showed that the LSTM classification accuracy was 15% higher than regular ANNs. Other studies using AI-based solar radiation prediction have also shown that LSTM has superior accuracy in predicting solar radiation. In this study by comparison, the classification accuracy was improved using LSTM, resulting in more effective use of time series data, once again confirming the effectiveness of this method. Comparisons with similar studies have also been added in the Discussion section. (Discussion, P.19-20, lines 319-324)

**Comment 7:**

Discuss about the limitations and future scope of the current work.

**Response:**

Thank you for your comments. This study has a few limitations. First, the echocardiograms were performed at the same institution. Second, this classification method is performed offline; therefore, it is necessary to apply it to real-time processing so that classification during echocardiography can be utilized. (Regarding as limitations and future works, P.22-23, lines 362-364)

We sincerely appreciate your comments and help for improving our manuscript. We hope that the revised manuscript is acceptable for publication.
