# Supplementary material for: Automated detection scheme for acute myocardial infarction using convolutional neural network and long short-term memory
Source: PLoS One. 2022 Feb 25;17(2):e0264002. doi: 10.1371/journal.pone.0264002 (PMC8880846; doi:10.1371/journal.pone.0264002)
Supplement: Attachment — Submitted filename: PLOSONE_response.docx [file pone.0264002.s002.docx]

PONE-D-21-16074
Title: Automated detection scheme for acute myocardial infarction using convolutional neural network and long short-term memory

**Dear Editor and Reviewers:**

Thank you for reviewing our manuscript. We have revised it based on your comments. Our responses are provided below.

**Response to Reviewer#3**

**Response to Reviewer#4**

**Comment1:**

The main limitation of this study is only one segment (anteroseptal infarction) was included, which may not be clinically applicable. Further, the authors included the myocardial infarction with proximal left anterior descending (LAD) artery occlusion which normally results in multiple abnormal segments. I am wondering that the authors take other segments into the account.

**Response:**

Thank you for your comment. In this study, we did not evaluate each segment of the heart individually; rather, we examined only acute anterior wall septal infarcts with occlusion of #6 in the American Heart Association Committee Report, which is the most frequent occurrence. Since this study focused only on acute anteroseptal infarction, we believe that the classification and evaluation of infarcts in each segment and in other coronary dominant regions should be performed in the future. These explanations have been added to the Discussion section. (Limitations and future works, P. 26, Lines 387-392)

**Comment2:**
Could the authors provide the criteria for myocardial infarction with proximal LAD occlusion in this study? Was coronary angiography performed for all patients to confirm the proximal LAD lesions?

**Response**

Thank you for your comment. All patients with anteroseptal infarction in this study underwent coronary angiography, which revealed the occlusion of #6. Patients who underwent percutaneous coronary intervention after coronary angiography and had abnormal wall motion on echocardiography were included. The diagnostic criteria for cases of acute myocardial infarction used in this study have been added to the Echocardiography subsection of the Materials and Methods section accordingly. (Echocardiography, P. 6, Lines 111-114)

**Commnet3:**
It would be much better if the authors can provide the time of echocardiography captured: is it before or after revascularization? Noticeably, echocardiograms were obtained after revascularization, the regional wall motion abnormalities may return to normal.

**Response:**

Thank you for your comment. The echocardiography used in this study, in which an anteroseptal infarction was confirmed, was performed after percutaneous coronary intervention. As you pointed out, we believe that the best timing for taking the view is shortly after symptom onset but before the initial percutaneous coronary intervention. However, just before percutaneous coronary intervention, the patient is in a high state of urgency, and the echoes taken may not have been recorded. In addition, the purpose of this study was to detect abnormal wall motion on echocardiography in patients with acute anteroseptal infarction. Therefore, here we performed echocardiography after percutaneous coronary intervention after confirming the occlusion of #6 by coronary angiography among selected patients who had abnormal wall motion in the anterior wall septum on echocardiography. Moreover, we classified abnormal wall motion due to anteroseptal infarction and normal myocardium without abnormal wall motion by inputting the results into deep learning models. These explanations have been added to the Echocardiography subsection of the Materials and Methods section accordingly. (Echocardiography, P. 6, Lines 111-114)

**Comment4:**
Most importantly, the occlusion of proximal LAD will result in multiple abnormal segments which are easily detected by echocardiogram in clinical practice. I am wondering how significantly this study contributes to clinical practice.

**Response**

Thank you for your comment. As you pointed out, specialists and skilled doctors can easily detect an anteroseptal infarction. However, it may be difficult for residents and physicians who are unfamiliar with echocardiography at the time of the initial visit or physicians in non-cardiology clinics to detect it. Anteroseptal infarction occurs frequently and requires accurate detection and diagnosis regardless of the technician or physician’s experience, field, or situation. This method can contribute to the detection of acute myocardial infarction and is expected to lead to the appropriate treatment of patients with acute myocardial infarction and improve their prognosis. Another technical novelty of this study is the use of LSTM, which enables a time-series analysis of wall motion on echocardiography. The results showed that LSTM can detect acute myocardial infarction more accurately than ANNs without a time-series analysis function, confirming its superiority using LSTM. In addition, here we used left ventricular long- and short-axis views (papillary muscle level), which are minimal and easy to depict for diagnosis, as input. Since the classifications were performed with highly accurate views, we found it possible to apply this method using LSTM to views other than the left ventricular long- and short-axis views (at the papillary muscle level), which are easy to image. In addition, although this study focused only on acute anteroseptal infarction, it is expected to be extended to detect infarcts in other coronary artery dominant regions using this method. This information is described in the Conclusion section.

(Conclusion, P. 26-27, Lines 397-411)

**Comment5:**
As a clinical physician, I would like to know the sensitivity, specificity, and area under the curve of this technique. These metrics will provide physicians deeper insights into this technique.

**Response:**

Thank you for your comment. The sensitivities, specificities, and AUCs are shown below. These results have been added to the Results section. (Results, P. 19-20, Lines 295-309)

Table7：Results of long-axis view images

| **View** | **Classifier** | **Input parameters**  **for the classifier** | **sensitivity** | **specificity** | **AUC** |
| --- | --- | --- | --- | --- | --- |
| **LX** | **LSTM** | **Output of FC2** | 0.838 | 0.864 | 0.870 |
|  |  | **GAP** | 0.879 | 0.913 | 0.943 |
|  |  | **GMP** | 0.889 | 0.901 | 0.871 |
|  | **ANN** | **Output of FC2** | 0.788 | 0.757 | 0.737 |
|  |  | **GAP** | 0.788 | 0.845 | 0.871 |
|  |  | **GMP** | 0.768 | 0.864 | 0.830 |

Table8：Results of short-axis papillary muscle view images

| **View** | **Classifier** | **Input parameters**  **for the classifier** | **sensitivity** | **specificity** | **AUC** |
| --- | --- | --- | --- | --- | --- |
| **PM** | **LSTM** | **Output of FC2** | 0.798 | 0.864 | 0.883 |
|  |  | **GAP** | 0.879 | 0.854 | 0.943 |
|  |  | **GMP** | 0.808 | 0.825 | 0.891 |
|  | **ANN** | **Output of FC2** | 0.777 | 0.825 | 0.796 |
|  |  | **GAP** | 0.717 | 0.874 | 0.901 |
|  |  | **GMP** | 0.818 | 0.854 | 0.869 |

**Fig 12. ROC curves of the LSTM versus ANN in long-axis view**

**Fig 13. ROC curves of the GAP versus GMP in long-axis view**

**Fig 14. ROC curves of the LSTM versus ANN in short-axis papillary muscle view**

**Fig 15. ROC curves of the GAP versus GMP in short-axis papillary muscle view**

**Comment6**
Lastly, it would suggest that the authors provide Table 1: baseline clinical characteristics of this cohort which will illustrate the target population that this technique is applying on.

**Response:**

Thank you for your comment. This table is presented below. This table has been added to the Echocardiography subsection of the Materials and Methods section. (Echocardiography, P. 8, Lines 136-138)

Table1: Baseline clinical characteristics of this cohort.

| Characteristic | Control  (n=103) | AMI  (n=99) | P value |
| --- | --- | --- | --- |
| **Age, mean (SD)** | 66.7(12.1) | 69.4 (11.6) | 0.21 |
| **Female sex, no. (%)** | 38(38.4) | 24 (24.2) | 0.051 |
| **Body mass index (kg/m^2^), mean (SD)** | 22.4(4.2) | 21.3(7.9) | 0.07 |
| **Systolic blood pressure (mmHg), mean (SD)** | 127.3(21.5) | 149.9 (29.6) | <0.001 |
| **Diastolic blood pressure (mmHg), mean (SD)** | 75.23(13.9) | 88.6 (20.8) | <0.001 |
| **Cardiac rhythm** | 76.7(13.5) | 84.1 (19.9) | 0.026 |
| **Sinus rhythm, mean (SD)** |  | 83.1 (21.6) |  |
| **Atrial fibrillation, mean (SD)** |  | 85.1 (20.6) |  |
| **Site of AMI** |  |  |  |
| **Anterior/lateral/inferior (%)** |  | Anterior (100) |  |
| **Coronary risk factors** |  |  |  |
| **Hypertension, no. (%)** | 4(4.04) | 58 (58.6) | <0.001 |
| **Diabetes, no. (%)** |  | 30 (30.3) |  |
| **Dyslipidemia, no. (%)** |  | 47 (47.5) |  |
| **Smoking, no. (%)** |  | 57 (57.6) |  |
| **Laboratory data** |  |  |  |
| **White blood cell count, mean (SD)** | 6.7(3.5) | 10.1 (7.0) | <0.001 |
| **Hemoglobin (g/dL), mean (SD)** | 10.8(2.5) | 13.7 (12.9) | <0.001 |
| **Creatinine (mg/dL), mean (SD)** | 1.2(1.7) | 1.2(1.3) | <0.001 |
| **eGFR (mL/min/1.73 m^2^), mean (SD)** | 78.4(32.1) | 82.3 (36.6) | 0.41 |
| **BNP (pg/mL), mean (SD)** | 85.8(144) | 391.8 (738.6) | 0.009 |
| **CK-MB (ng/ml), mean (SD)** | 5.8(5.9) | 280.9 (452.1) | <0.001 |
| **TnI (ng/ml), mean (SD)** | 0.2(0.6) | 89.3 (226.1) | <0.001 |
| **Medications** |  |  |  |
| **β-blocker, no. (%)** | 16(15.1) | 63 (63.6) | <0.001 |
| **ARB /ACE-I, no. (%)** | 8(7.8) | 65 (65.6) | <0.001 |

Figure3**: Selection of study population**
